# Long non-coding RNA CASC7 suppresses malignant behaviors of breast cancer by regulating miR-21-5p/FASLG axis

**DOI:** 10.1080/21655979.2021.2010372

**Published:** 2021-12-10

**Authors:** Genjin Wang, Peng Duan, Feng Liu, Zhengkuo Wei

**Affiliations:** a Department of General Surgery, Section IV, Xiangyang No. 1 People’s Hospital, Hubei University of Medicine, Xiangyang, China; b Department of Obstetrics and Gynaecology, Xiangyang No. 1 People’s Hospital, Hubei University of Medicine, Xiangyang, China

**Keywords:** lncRNA CASC7, miR-21-5p, FASLG, breast cancer

## Abstract

Recently, it has been increasingly proved that lncRNAs are functionally involved in a majority of tumor progression. LncRNA CASC7 has also been revealed to participate in the development of several cancers as a tumor promoter or suppressor. Herein, we focus on uncovering the role and underlying molecular mechanism of CASC7 in breast cancer. Tumor tissues and the paired paracancerous tissues from the breast cancer patients were used to evaluate the level of CASC7 in breast cancer. By analyzing the CASC7 expression in breast cancer cell lines, both the expression levels of CASC7 in cancer tissues and cell lines were obviously downregulated compared to those in paired paracancerous tissues and normal human epithelial MCF10A cells. Subsequently, the construction of lentivirus overexpression system (oe-CASC7 and oe-NC) was used to elevate the expression of CASC7. A series of functional experiments were conducted to show that the cell proliferation, migration, and invasion were inhibited when CASC7 overexpressed in breast cancer cells. Meanwhile, the apoptosis of oe-CASC7 cells was induced compared to the oe-NC breast cancer cells. We further confirmed that CASC7 functions by regulating miR-21-5p/FASLG axis. Finally, a xenograft model in nude mice verified that CASC7 was a tumor suppressor in breast cancer. These results suggest that lncRNA CASC7 suppresses to malignant behaviors of breast cancer by modulating miR-21-5p/FASLG axis. **Abbreviations** lncRNAs: long non-coding RNAs; ceRNA: competing endogenous RNA; CASC7: cancer susceptibility candidate 7; miRNAs: MicroRNAs; MAPK10: mitogen-activated protein kinase 10; FASLG: Tumor Necrosis Factor Ligand Superfamily Member 6; FAS: Tumor Necrosis Factor Receptor Superfamily Member 6

## Introduction

The incidence of breast cancer is continuously rising year by year [1,2]. Nowadays, breast cancer, as the most prevalent malignant cancer among females, is the first leading cause of cancer death in women [2]. Not only breast cancer endangers the life quality of patients but also the large population of patients with breast cancer has put tremendous pressure on public health [3]. Notably, the specific molecular mechanisms of occurrence and progression of breast cancer remain unclear [4]. Therefore, excavating a potential therapeutic molecule in breast cancer is urgently needed [4].

LncRNAs (long non-coding RNAs) are RNA transcripts without protein coding, which are longer than 200 nucleotides in length [5]. Emerging evidence has revealed that numerous lncRNAs implicated directly or indirectly in the development of most cancers [5,6]. Also, in breast cancer, it is reported that several lncRNAs accelerate or retard tumorigenesis and progression [5,7]. Hence, lncRNAs could be an attractive therapeutic target in breast cancer [7]. Among the many lncRNAs, lncRNA CASC7 (cancer susceptibility candidate 7) is an RNA gene and is affiliated with the lncRNA class [8]. It has been previously reported that lncRNA CASC7 functions as a tumor suppressor in both non-small-cell lung cancer and thyroid cancer [9,10]. Besides, lncRNA CASC7 is associated with several pathologies, such as heart failure and severe asthma [11,12]. Nevertheless, the role of CASC7 has not been unraveled in breast cancer. Thus, it is necessary to clarify how CASC7 performs its function in breast cancer.

In general, lncRNAs sponge miRNAs to regulate the downward target expression following the ceRNA (competing endogenous RNA) regulation network [13]. Once dysregulation of the specific lncRNA occurs, the related miRNAs get aberrant in expression and, subsequently, influence the expression of the target protein post-transcriptionally [13,14]. At the cellular levels, the intracellular signaling pathways are affected as well as the cell malignant behaviors when the lncRNAs rise an abnormal expression pattern [14–16]. To sum up, it is considered that the lncRNAs usually contribute to their functional role by the downward specific miRNA and protein axis.

MiR-21-5p, as a kind of small non-coding RNA molecules (21–23 nucleotides), takes an important part in the biological processes [17]. Among the most cancers, miR-21-5p commonly presents an upregulated trend [18–20]. A large number of studies indicated that miR-21-5p acts an oncogenic factor including lung cancer and ovarian cancer [20,21]. Moreover, in breast cancer, it is pointed that miR-21-5p enhances breast cancer progression by modulating the mitogen-activated protein kinase10 (MAPK10) [22]. In addition, FASLG (Tumor Necrosis Factor Ligand Superfamily Member 6) is a member of the tumor necrosis factor superfamily, which encoded transmembrane protein to induce apoptosis triggered by binding to FAS (Tumor Necrosis Factor Receptor Superfamily Member 6) [23]. Due to FASLG/FAS signaling could induce apoptosis commonly in various cancers, the FASLG is summarized as a tumor suppressor [23–25]. However, these interactions between lncRNA CASC7 and miR-21-5p or miR-21-5p and FASLG are still indefinite in breast cancer.

To recapitulate, we proposed a hypothesis that lncRNA CASC7 is associated with the breast cancer development by regulating miR-21-5p/FASLG axis. Our aim is to reveal the role of lncRNA CASC7 and investigate these interactions in human breast cancer.

## Materials and methods

### Breast cancer tissues

The 26 female breast cancer patients’ tumor tissues and paired paracancerous tissues were obtained from Xiangyang No. 1 People’s Hospital between 2019 and 2021. All patients provided written informed consent. This study was reviewed and approved by the Ethics Review Committee of Hubei University of Medicine and was performed in accordance with Declaration of Helsinki. The clinicopathological characteristics of breast cancer patients are shown in Table 1.

**Table 1.** The clinicopathological characteristics of 26 breast cancer patients

Characteristics	Numbers
Age	
≤50	8
>50	18
Lymph node mestasis	
Yes	16
No	10
Pathological staging	
I + II	11
III + IV	15
Distant metastasis	
M0	12
M1	14
Tumor subtypes	
ER+/PR+	11
ER+/PR-	7
Her2+	6
Triple negative	2

ER, estrogen receptor; PR, progesterone receptor. Low/high by the sample mean.

### qRT-PCR

The tissues and cells were extracted to RNA and then reversed to cDNA for conducting qRT-PCR assay by TRIzol reagent and reverse transcription kit (Invitrogen). Actin and U6 were used as reference genes for mRNA and miRNA, respectively. qRT-PCR analysis was evaluated by a CFX96 real-time PCR system with SYBR (TaKaRa, China). The relative expressions were calculated by using 2^−ΔΔCt^ method. The primers are listed in Table 2.

**Table 2.** The list of primers

Name	Forward/reverse	Sequence (5ʹ to 3ʹ)
CASC7	F	AACATGGTCTCTTGGTGCCTGATG
	R	CCACGGTAAGCGACGAGGAATC
miR-21-5p	F	GCTTATCAGACTGATGTTG
	R	GAACATGTCTGCGTATCTC
FASLG	F	TGCCTTGGTAGGATTGGGC
	R	GCTGGTAGACTCTCGGAGTTC
actin	F	CATGTACGTTGCTATCCAGGC
	R	CTCCTTAATGTCACGCACGAT
U6	F	CTCGCTTCGGCAGCACA
	R	AACGCTTCACGAATTTGCGT

### Cells culture

The DMEM medium with 10% FBS, 100 IU/mL penicillin, and 100 μg/mL streptomycin (Gibco, the US) was used to culture the human breast cancer cell lines, T47D, MCF-7 and MDA-MB-231. Human normal breast basal epithelial cells (MCF10A) were maintained in 50/50 DMEM/F12 (Gibco) media supplemented with 5% horse serum, insulin, EGF, cholera toxin and hydrocortisone. All these cells were obtained from Cell Bank of Type Culture Collection of Chinese Academy of Sciences (Shanghai, China) and cultured at 37°C, 5% CO_2_.

### Cell transfections

The lentivirus system (oe-CASC7 and oe-NC, MOI = 50) was used to infect the breast cancer cells to stably overexpression CASC7. Compared with the overexpression of whole full-length CASC7 lentiviral production (oe-CASC7), oe-NC is an empty lentiviral vector used as a control. Both oe-CASC7 and oe-NC lentiviral vector production are obtained from Genechem, Shanghai, China. Oe-CASC7 and oe-NC breast cancer cells were collected after incubating with 1 mg/ml puromycin for 72 h. About at 70% confluence, cells were transfected with miR-21-5p mimic (100 pmol) and corresponding negative control (100 pmol, Invitrogen, Shanghai, China) by Lipofectamine™2000 (ThermoFisher, USA) to conduct follow-up experiments.

### Cell viabilities

2000 transfected cells were planted into 96-well plates per well. After the indicated time points, 10 μl CCK-8 (Beyotime, Shanghai, China) was added into the well for another 4 h to measure cell viabilities. The results were detected by a Microplate Reader (Bio-Rad, USA) at 450 nm.

### Colony formation assays

500 cells were evenly planted into a 6-well plate. After 12 days, the cells were stained with crystal violet and counted the colony numbers (>50 cells were identified as a colony).

### Migration and invasion

For migration and invasion, the transwell chamber (8 µm, CorningLife Sciences, Corning, NY, USA) was used to detect cell migratory cells, and the transwell chamber with 50 μl Matrigel gel was used to detect cell invasive cells. 20,000 cells were seeded into the upper chamber with 100 μl of serum-free medium, and 500 μl of DMEM medium containing 20% serum was added to the lower chamber. After 24 h of incubation, the cells attached to the lower surface of the upper chamber were stained with crystal violet and analyzed under a microscope.

### Apoptotic cells

The apoptotic cell rates were analyzed by flow cytometry (FACScan, Beckman Coulter, USA) with an Annexin V-FITC/PI (Invitrogen, USA) assay. Briefly, all supernatant and adherent cells were collected 48 h after transfection for apoptotic staining. The apoptotic cell suspension was prepared and operated in accordance with the instructions of apoptosis detection kits.

### Luciferase reporter assays

All plasmids were obtained from GenePharma, China. The corresponding sequenceis inserted into a luciferase report gene vectors (pRL-TK, Promega). The reporter plasmids and miR-21-5p mimic or miR NC were co-transfected into cells for 48 h. The relative luciferase activity was detected by Dual-Luciferase Reporter Assay System (Promega).

### Western blotting

Total protein is lysed by RIPA Lysis Buffer (Beyotime, Shanghai, China) and denatured with loading buffer to load into 10% SDS-PAGE gels. Subsequently, the samples were separated and transferred to PVDF membrane (Bio-Rad, USA). Then, the primary antibody, anti-FASLG (1:1000, # PA1576, BosterBio), was incubated after blocking with blocking buffer overnight. On the second day, HRP conjugated secondary antibodies were incubated after washing. The results were examined by chemiluminescence. A reference anti-actin (1:1000, ab8245, Abcam) was used to internal reference.

### Animal experiment

The total 10 nude female mice (6-week old) were randomly divided into two groups to conduct animal experiment. On day 0, we inoculated 6 × 10^6^ oe-NC (*n* = 5) and oe-CASC7 (*n* = 5) MCF-7 cells on the flank of nude mice. Every 3 days, the tumor volume according to volume = 1/2 × length × width^2^. On day 15, we weighted the tumors and then fixed in paraformaldehyde.

### Immunohistochemistry (IHC)

The 4-μm tumor sections were first deparaffinized and then incubated with H_2_O_2_ to eliminate endogenous peroxides. After blocking with blocking solution, the sections were incubated with antibodies, anti-FASLG (1:200, # PA1576, BosterBio) or anti-Ki67 antibody (1:100, ab15580, Abcam) at 4°C overnight. On day 2, the sections were washed to incubate secondary antibody and HRP (Beijing Zhongshan Golden Bridge Biotechnology Co., Ltd) and then incubate with 3,3′-diaminobenzidine (DAB, Sigma-Aldrich, St Louis, MO, USA) for 3 min to exhibit the positive marker. IHC images were taken by an light microscope (Olympus).

### Statistical analysis

All statistical analyses were conducted using the SPSS statistical package (16.0, SPSS Inc., Chicago, IL). Unpaired Student’s *t* test was used to compare the means of two groups of data. The data were shown as mean ± SD. *P*-values were calculated using ANOVA, with *P* <0.05 considered as significant.

## Results

Our work focuses on uncovering the role and underlying molecular mechanism of CASC7 in breast cancer. We first investigated that the expression of lncRNA CASC7 is downregulated and concluded its role as a tumor suppressor in breast cancer. To make clear how lncRNA CASC7 exerts its function in breast cancer, we subsequently performed rescue experiments to illustrate that lncRNA CASC7 suppresses to malignant behaviors of breast cancer by regulating miR-21-5p/FASLG pathway.

### LncRNA CASC7 is characterized as a tumor suppressor in breast cancer

**Figure 1.**
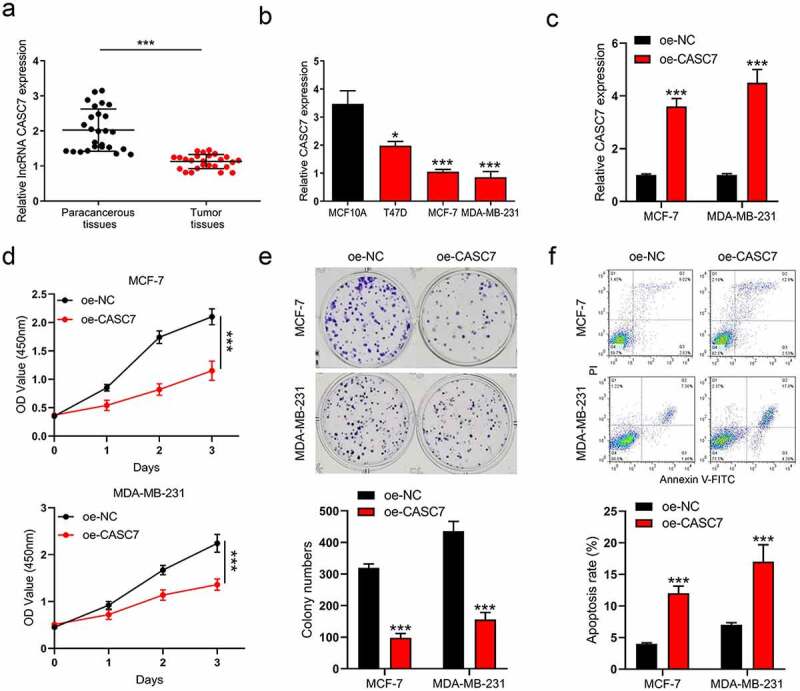
LncRNA CASC7 is characterized as a tumor suppressor in breast cancer. (a) The level of CASC7 in breast tumor tissues and paracancerous tissues was examined by RT-qPCR. (b) The levels of CASC7 were examined by RT-qPCR in MCF10A and breast cancer cells. (c) The expression of CASC7 was detected by qRT-PCR. (d) The cell viability was detected by CCK-8 assay in MCF-7 and MDA-MB-231 cells. (e) The colony formation was examined in MCF-7 and MDA-MB-231 cells. (f) The apoptosis was examined in MCF-7 and MDA-MB-231 cells. Data are presented as mean ± SD, *n* = 3. *P < 0.05, ***P < 0.001

To determine the expression pattern of CASC7 in breast cancer, 26 pairs of collected tumor tissues and the paired paracancerous tissues were used to detect the level of CASC7. The expression of CASC7 was inhibited in tumor tissues compared to paracancerous tissues (Figure 1(a)). Meanwhile, we further detected the CASC7 expression in breast cancer cell lines (T47D, MCF-7 and MDA-MB-231) and the normal human epithelial MCF10A cells. The result pointed out that the expressions of CASC7 were also downregulated in breast cancer cells compared to MCF10A cells (Figure 1(b)). Both the expression levels of CASC7 were hindered in cancer tissues and cells, and we consequently constructed a lentivirus overexpression system (oe-CASC7 and oe-NC) to elevate the expression of CASC7 (Figure 1(c)). Then, we intended to explore whether boosting the expression of CASC7 could affect the malignant behaviors of breast cancer. The result showed that overexpressing CASC7 decreased the cell viability of breast cancer cells compared to oe-NC cells by a CCK-8 assay (Figure 1(d)). Meanwhile, the colony formation assays exhibited that the clonogenic abilities of breast cancer cells were restricted by boosting CASC7 expression (Figure 1(e)). Apart from the proliferation of breast cancer cells was influenced, in Figure 1(f), CASC7 increased the number of apoptotic cells. To sum up, CASC7 restricted the proliferation of the two breast cancer cells and induced cell apoptosis. Thus, CASC7 is characterized as a tumor suppressor in breast cancer cells.

### Overexpression of lncRNA CASC7 inhibits the migration and invasion of breast cancer cells

**Figure 2.**
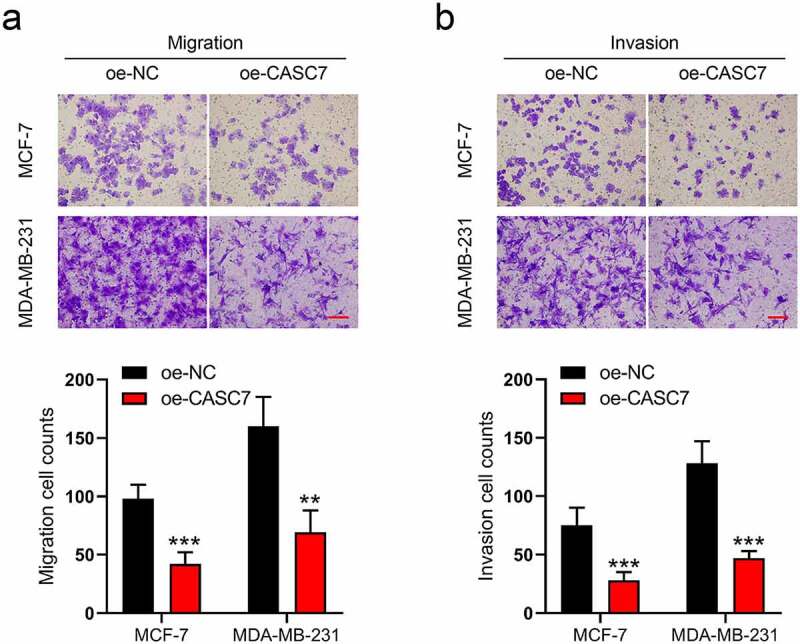
Overexpression of lncRNA CASC7 inhibits the migration and invasion of MCF-7 and MDA-MB-231 cells. (a) The migration was examined in MCF-7 and MDA-MB-231 cells. (b) The invasion was examined in MCF-7 and MDA-MB-231 cells. Scale bar: 100 µm. Data are presented as mean ± SD, *n* = 3. **P < 0.01, ***P < 0.001

Moreover, in MCF-7 and MDA-MB-231 cells, the migration and invasion cells were also affected by CASC7 overexpression. As shown in Figure 2(a), the numbers of migrated cells decline in oe-CASC7 cells compared to the oe-NC cells. Similarly, boosting the CASC7 expression decreased the numbers of invasive cells as well (Figure 2(b)). According to the above results, we revealed that the overexpression of lncRNA CASC7 inhibits the migration and invasion of breast cancer cells.

### MiR-21-5p is regulated by lncRNA CASC7

**Figure 3.**
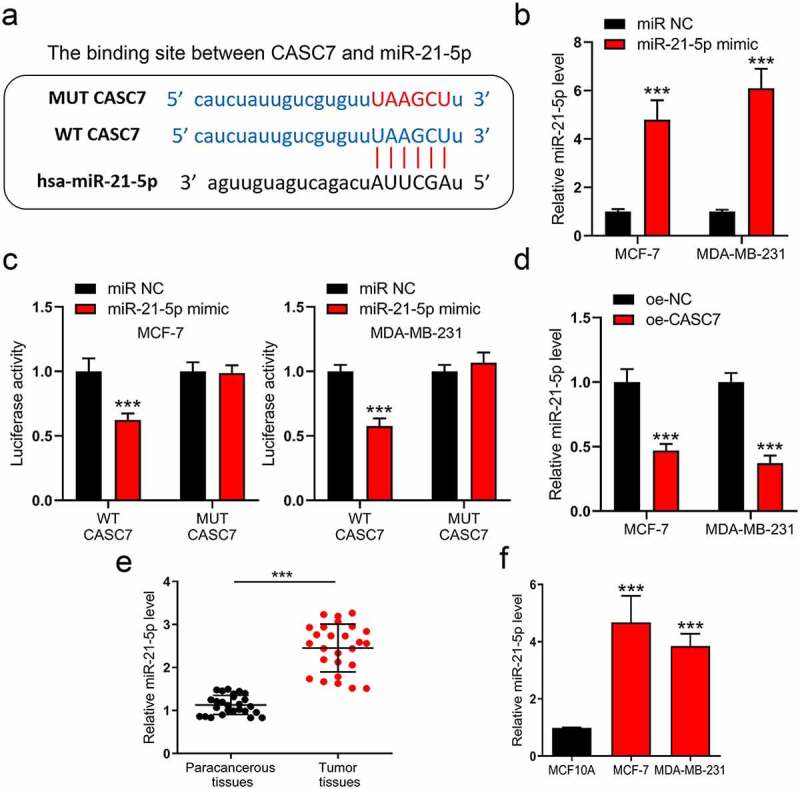
MiR-21-5p is regulated by lncRNA CASC7 in MCF-7 and MDA-MB-231 cells. (a) The binding site between miR-21-5p and CASC7. (b) The levels of miR-21-5p were examined by RT-qPCR in MCF-7 and MDA-MB-231 cells. (c) The luciferase assay was examined in MCF-7 and MDA-MB-231 cells. (d) The levels of miR-21-5p were examined by RT-qPCR in MCF-7 and MDA-MB-231 cells. (e) The level of miR-21-5p in breast tumor tissues and paracancerous tissues was examined by RT-qPCR. (f) The levels of miR-21-5p were examined by RT-qPCR in MCF10A, MCF-7 and MDA-MB-231 cells. Data are presented as mean ± SD, *n* = 3. ***P < 0.001

Since lncRNAs commonly play their roles through a ceRNA network, we consequently analyzed the downward potential miRNAs of CASC7. By excavating the underlying binding site on the online database (DIANA-LncBase), miR-21-5p is a potential target of CASC7. The binding site is shown in Figure 3(a). To verify whether lncRNA CASC7 could regulate the miR-21-5p expression, luciferase reporter plasmids were used to examine the interaction between lncRNA CASC7 and miR-21-5p. Before conducting this experiment, we first validated that miR-21-5p mimic could amplify miR-21-5p expression (Figure 3(b)). Subsequently, the cells were co-transfected with miR-21-5p mimic and wild-type luciferase plasmid or mutant luciferase plasmid. MiR-21-5p mimic inhibited the luciferase activity of WT plasmid without affecting the MUT plasmid (Figure 3(c)). Therefore, we investigated miR-21-5p degree in oe-NC and oe-CASC7 breast cancer cells. As shown in Figure 3(d), CASC7 inhibits miR-21-5p expression obviously. Hence, miR-21-5p is downregulated by lncRNA CASC7 in MCF-7 and MDA-MB-231 cells. Meanwhile, miR-21-5p content was also detected in breast cancer tissues and cells. Both miR-21-5p expressions were augmented in breast cancer tissues and cells (Figure 3(e,f)).

### MiR-21-5p regulates FASLG expression

**Figure 4.**
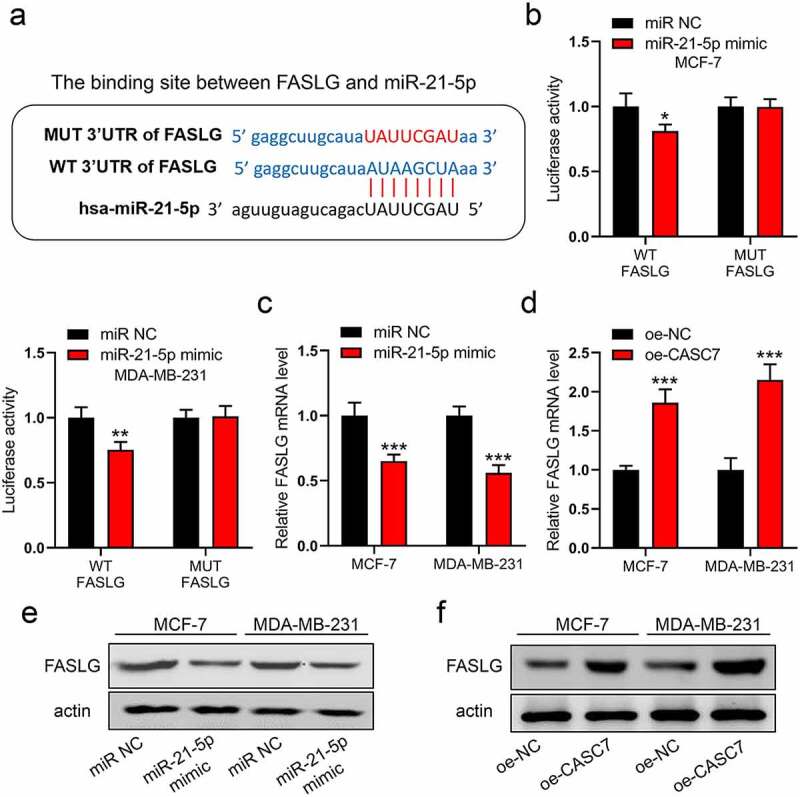
MiR-21-5p regulates the expression of FASLG. (a) The binding site between miR-21-5p and FASLG. (b) The luciferase assay was examined in MCF-7 and MDA-MB-231 cells. (c) and (d) The mRNA expressions of FASLG were examined by RT-qPCR. (e) and (f) The protein expressions of FASLG were examined by Western blotting. Data are presented as mean ± SD. *n* = 3, **P < 0.01, ***P < 0.001

To continuously excavate the downward target of miR-21-5p, FASLG is a potential target with high scoring on an online database (http://mirdb.org). The binding site between miR-21-5p and FASLG is shown in Figure 4(a). Similarly, we validated this interaction by luciferase assay. In Figure 4(b), miR-21-5p mimic only decreased the breast cancer cells transfected with WT plasmid, while it did not impact the cells with MUT plasmid. Furthermore, miR-21-5p mimic could inhibit FASLG expression in mRNA and protein levels (Figure 4(c,e)). However, CASC7 overexpression enhanced directly the mRNA and protein levels of FASLG (Figure 4(d,f)). Thus, our data indicated that both the miR-21-5p and CASC7 could regulate FASLG expression in breast cancer cells.

### LncRNA CASC7 suppresses malignant behaviors by regulating miR-21-5p/FASLG axis in breast cancer

**Figure 5.**
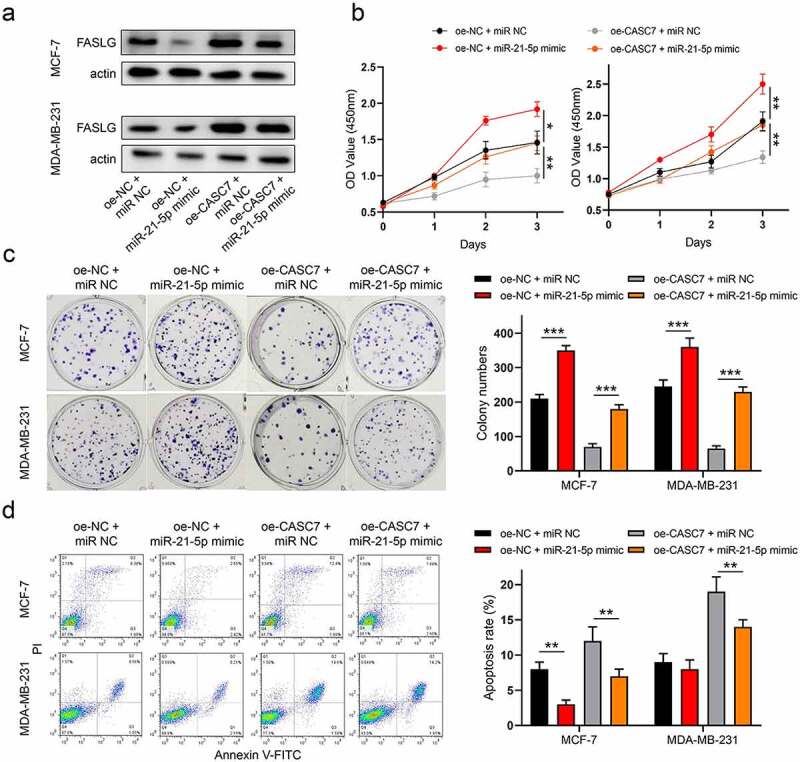
LncRNA CASC7 contributes to malignant behaviors of breast cancer by regulating miR-21-5p/FASLG axis. (a) The protein expressions of FASLG were examined by Western blotting. (b) The cell viability was detected by CCK-8 assay. (c) The colony formation was examined. (f) The apoptosis was examined. Data are presented as mean ± SD. *n* = 3, **P < 0.01, ***P < 0.001

To uncover the underlying mechanism of CASC7 regulates the development of breast cancer, a series of rescue experiments was used to uncover how CASC7 exerts its biological functions. As in Figure 5(a), miR-21-5p mimic mitigated the increment of FASLG expression by CASC7 overexpression in breast cancer cells. At the same time, the inhibition effect of CASC7 overexpression on cell proliferation was abrogated by miR-21-5p mimic in two cancer cells (Figure 5(b,c)). Likewise, miR-21-5p mimic relieved the facilitation effect of CASC7 overexpression on apoptosis (Figure 5(d)). As a consequence, we confirmed that lncRNA CASC7 regulates miR-21-5p/FASLG axis to suppress the development of breast cancer.

### A xenograft model revealed that overexpression of lncRNA CASC7 inhibits breast cancer development

**Figure 6.**
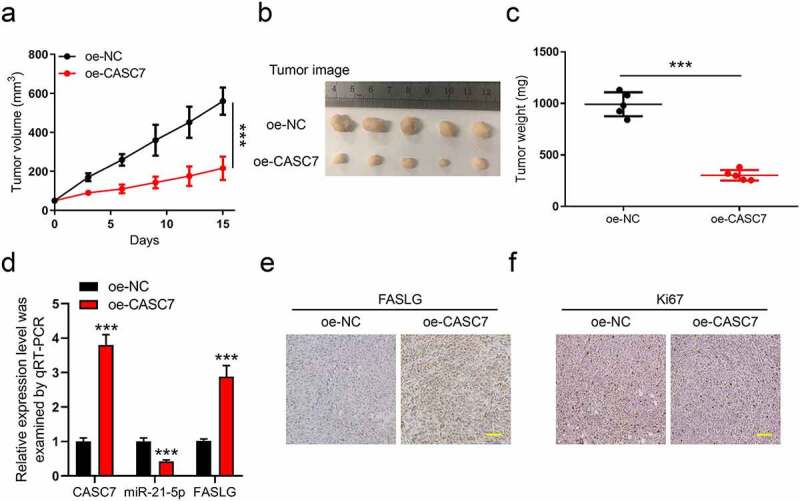
A xenograft model revealed that overexpression of lncRNA CASC7 inhibits the progression of breast cancer. The total 10 Balb/c nude mice were divided into two groups (oe-CASC7, *n* = 5, and oe-NC, *n* = 5). (a) The tumor volume was recorded every 3 days. (b) The images of tumors were taken on day 15. (c) The tumor weight was weighted after tumors were stripped. (d) The levels of CASC7, miR-21-5p, and FASLG in tumors were analyzed by qRT-PCR. (e) The FASLG level in tumors was analyzed by IHC. (f) The Ki67 levels in tumors were analyzed by IHC. Scale bar: 50 µm. Data are presented as mean ± SD. **P < 0.01, ***P < 0.001

Finally, a xenograft model with oe-CASC7 and oe-NC tumors was used to verify our above results. The total 10 Balb/c nude mice were divided into two groups (oe-CASC7, *n* = 5, and oe-NC, *n* = 5). On day 15 after inoculation, we collected the tumors for weighting and subsequent experiments. During the 15 days, we recorded the tumor volume every 3 days (Figure 6(a)). The tumor image and tumor weight are shown in Figure 6(b,c), respectively. This leads one to believe that CASC7 overexpression restricted both the tumor growth and weight obviously. Moreover, in oe-CASC7 groups, the qPCR analysis indicated that the expression of CASC7 and FASLG was upregulated and miR-21-5p was inhibited, respectively (Figure 6(d)). Also, in Figure 6(e), the IHC results confirmed that the overexpression of CASC7 elevated the protein level of FASLG in tumors. In addition, oe-CASC7 lowered the expression of ki67 while elevated the FASLG level in the tumor (Figure 6(f)). In all, our study proved that overexpression of lncRNA CASC7 inhibits the progression of breast cancer.

## Discussion

In our work, we first reported that lncRNA CASC7 was a tumor suppressor in breast cancer. Meanwhile, a series of experiments showed that CASC7 overexpression inhibited breast cancer cell proliferation as well as migration and invasion. In addition, the apoptosis of CASC7 could be induced by CASC7 in MCF-7 and MDA-MB-231 cells. Furthermore, we validated that LncRNA CASC7 suppressed to malignant behaviors of breast cancer by modulating miR-21-5p/FASLG axis. This inhibition effect of CASC7 on breast cancer was also verified by an MCF-7 xenograft model. In all, we illustrated that lncRNA CASC7 suppresses breast cancer progression.

Recently, several studies have revealed the role of CASC7 in other cancers and pathologies. It was reported that CASC7 retarded the progression of multiple cancers, such as papillary thyroid carcinoma, neuroblastoma, glioma and non-small-cell lung cancer [9,10,26,27]. All these researches uniformly pointed out that lncRNA CASC7 is a cancer suppressor. Consistently, our data suggesting an anti-cancer role of CASC7 is in breast cancer. Also, in breast cancer, Banerjee et al. proposed that lncRNA CASC7 has been identified to be associated with the development and metastasis of breast cancer by the bioinformatic analysis [28]. Thus, we can conclude that CASC7 is involved in the progression of breast cancer as a tumor suppressor.

Similarly, it has been identified that miR-21-5p should be a cancer promoter in most cancers [29]. Studies have been reported that, in lung cancer, miR-21-5p promotes cell malignant behaviors by impairing SMAD7 expression [30]. Likewise, miR-21-5p inhibits mitogen-activated protein kinase 10 to promote the progression of breast cancer [22]. Interestingly, this is the reason that lncRNA CASC7 could sponge miR-21-5p to weaken the oncogenic effect of miR-21-5p. Furthermore, FASLG is closely associated with apoptosis in a large number of cancers [24,31]. Precisely, because of FASLG as a member of the tumor necrosis factor superfamily, the apoptosis of cells was directly initiated once FAS binding to FASLG [31]. For this reason, lots of researchers intend to activate the FAS/FASLG signaling for breast cancer treatment [31]. To sum up, miR-21-5p plays an oncogenic role and FASLG is a classical tumor suppressor in most cancer, respectively. Notably, our results suggested that lncRNA CASC7 plays its anti-cancer effect to inhibit miR-21-5p level, eventually amplifying the FASLG level in breast cancer.

## Conclusion

In summary, CASC7 was significantly repressed in breast cancer, and boosting the expression level of CASC7 retarded breast cancer progression. Finally, we validated that lncRNA CASC7 suppresses malignant behaviors of breast cancer by regulating the miR-21-5p/FASLG axis.
